# Structure of the CaMKIIδ/Calmodulin Complex Reveals the Molecular Mechanism of CaMKII Kinase Activation

**DOI:** 10.1371/journal.pbio.1000426

**Published:** 2010-07-27

**Authors:** Peter Rellos, Ashley C. W. Pike, Frank H. Niesen, Eidarus Salah, Wen Hwa Lee, Frank von Delft, Stefan Knapp

**Affiliations:** University of Oxford, Nuffield Department of Clinical Medicine, Structural Genomics Consortium, Oxford, United Kingdom; University of California San Diego, United States of America

## Abstract

Long-term potentiation (LTP), a long-lasting enhancement in communication between neurons, is considered to be the major cellular mechanism underlying learning and memory. LTP triggers high-frequency calcium pulses that result in the activation of Calcium/Calmodulin (CaM)-dependent kinase II (CaMKII). CaMKII acts as a molecular switch because it remains active for a long time after the return to basal calcium levels, which is a unique property required for CaMKII function. Here we describe the crystal structure of the human CaMKIIδ/Ca^2+^/CaM complex, structures of all four human CaMKII catalytic domains in their autoinhibited states, as well as structures of human CaMKII oligomerization domains in their tetradecameric and physiological dodecameric states. All four autoinhibited human CaMKIIs were monomeric in the determined crystal structures but associated weakly in solution. In the CaMKIIδ/Ca^2+^/CaM complex, the inhibitory region adopted an extended conformation and interacted with an adjacent catalytic domain positioning T287 into the active site of the interacting protomer. Comparisons with autoinhibited CaMKII structures showed that binding of calmodulin leads to the rearrangement of residues in the active site to a conformation suitable for ATP binding and to the closure of the binding groove for the autoinhibitory helix by helix αD. The structural data, together with biophysical interaction studies, reveals the mechanism of CaMKII activation by calmodulin and explains many of the unique regulatory properties of these two essential signaling molecules.

**Enhanced version:**

** This article can also be viewed as an enhanced version in which the text of the article is integrated with interactive 3-D representations and animated transitions. Please note that a web plugin is required to access this enhanced functionality. Instructions for the installation and use of the Web plugin are available in Text S1.**

## Abstract 2

Structural and biophysical studies reveal how CaMKII kinases, which are important for cellular learning and memory, are switched on by binding of Ca^2+^/calmodulin.

## Author Summary

CaMKII enzymes transmit calcium ion (Ca^2+^) signals released inside the cell by regulating signal transduction pathways through phosphorylation: Ca^2+^ first binds to the small regulatory protein CaM; this Ca^2+^/CaM complex then binds to and activates the kinase, which phosphorylates other proteins in the cell. Since CaMKs remain active long after rapid Ca^2+^ pulses have dropped they function as molecular switches that turn on or off crucial cell functions in response to Ca^2+^ levels. The multifunctional CaMKII forms of this enzyme – of which there are four in human – are important in many processes including signaling in neurons and controlling of the heart rate. They are particularly abundant in the brain where they probably play a role in memory. CaMKII forms an exceptionally large, dodecameric complex. Here, we describe the crystal structure of this complex for each of the four human CaMKII catalytic domains in their autoinhibited states, a complex of CaMKII with Ca^2+^/CaM, as well as the structure of the oligomerization domain (the part of the protein that mediates complex formation) in its physiological dodecameric state and in a tetradecameric state. Detailed comparison of this large body of structural data together with biophysical studies has allowed us to better understand the structural mechanisms of CaMKII activation by CaM and to explain many of the complex regulatory features of these essential enzymes.

## Introduction

Calcium/Calmodulin (Ca^2+^/CaM)-dependent serine/threonine kinases (CaMKs) constitute a family of 81 proteins in the human proteome that play a central role in cellular signaling by transmitting Ca^2+^ signals [1]. Kinases in this protein family are activated through binding of Ca^2+^/CaM to regulatory regions that either flank the catalytic domain or are located in regulatory molecules [2]. Four CaMKII isozymes (α, β, γ, and δ), in addition to about 30 splice variants, are expressed in humans. The α and β isoforms are brain specific and together make up approximately 1% of total brain protein in rodents and up to 2% of total protein in their hippocampus [3]. The γ and δ isoforms are expressed in most tissues, but in comparison have much lower expression levels [4],[5]. The unique switch-like properties of CaMKII activation and its extremely high abundance in the brain identified CaMKII as a key regulator of cellular memory and learning [6]. CaMKII is essential for the induction of long-term potentiation (LTP), a long-lasting increase in the efficiency of synaptic transmission between neurons that is believed to be a cellular correlate of memory [7],[8]. Stimuli that induce LTP lead to autophosphorylation at T286 in CaMKIIα (T287 in the β, γ, and δ isoforms), thereby resulting in sustained CaMKII activation [9]; mice expressing the CaMKIIα T286A mutant were severely impaired in learning [10].

Several CaMKIIδ variants are highly abundant in myocardial tissue [11],[12]. Increased CaMKII activity has been observed in patients with structural heart disease and arrhythmias, where prolonged action potential duration leads to sustained hyperactivation of CaMKII and heart failure [11].

CaMKII proteins form large oligomeric structures. The N-terminal kinase domain is tethered via an autoinhibitory helix and a calmodulin binding site to a C-terminal oligomerization domain that organizes the enzyme into ring-shaped oligomers. Three-dimensional reconstruction of single-particle electron microscopy images revealed dodecameric assemblies for all purified homogeneous full-length CaMKII isozymes [13],[14]. In contrast, tetradecamers were detected in the crystal structures of isolated oligomerization domains. This non-physiological oligomerization state has been attributed to the absence of the kinase domain [15],[16].

The cellular regulation of CaMKII activity is the outcome of a complex interplay between protein localization, heterooligomerization, local Ca^2+^/CaM concentrations, CaMKII autophosphorylation, and dephosphorylation of CaMKII by phosphatases [2],[17]. The structure of the isolated *Caenorhabditis elegans* CaMKII (CeCaMKII) kinase domain in its autoinhibited state provided the first insight into the molecular mechanism of CaMKII regulation [18]. In this structure the inhibitory domain forms a helix that binds tightly to the substrate binding pocket preventing access of substrates. Interestingly, the regulatory domains of two catalytic domains interacted as antiparallel coiled-coils in the CeCaMKII structure suggesting that the inhibitory helix mediates self association in the inactive state of the enzyme. This association model has also been evoked to explain cooperativity of CaMKII activation by Ca^2+^/CaM observed in enzyme kinetic assays and “pairing” of kinase domains in autoinhibited holoenzymes [19],[20]. In the inactive state the autophosphorylation site within the regulatory domains is not accessible [18]. It has been speculated that this inhibitory “block” of the regulatory domain is released by structural changes induced upon Ca^2+^/CaM binding. Once phosphorylated at the regulatory T286 site (CaMKIIα numbering) by catalytic domains present in the same holoenzyme, steric constraints prevent rebinding of the autoinhibitory domain to the catalytic domain [4],[21],[22].

In addition, CaMKII can be made insensitive to Ca^2+^/CaM by autophosphorylation at T305/T306 located within the Ca^2+^/CaM binding site [23],[24], a process that is facilitated by interaction with the membrane associated guanylate kinase (MAGUK/CASK) [25],[26]. The balance between the Ca^2+^/CaM-sensitive and -insensitive CaMKII pool is critical for the regulation of post-synaptic plasticity [27],[28].

In CaMKIIα, autophosphorylation of T306 but not of T305 was observed in vitro, leading to a strong reduction of Ca^2+^/CaM binding [29]. The region flanking this autophosphorylation site represents a non-consensus substrate site for CaMKII, which raises the question of how this motif would be efficiently recognized as a substrate.

To date, our structural knowledge of how CaMKIIs are activated is based solely on structures of isolated kinase domains and peptide complexes of either catalytic domains with their substrates or Ca^2+^/CaM with calmodulin binding sites [18],[20]. We were interested in describing the molecular mechanisms that govern CaMKII activation in an intact catalytic domain/Ca^2+^/CaM complex. The structure of the CaMKIIδ/Ca^2+^/CaM presented here captures the kinase in a state where the inhibitory helix is dislodged from the substrate binding site, thereby making it available for autophosphorylation by an adjacent kinase molecule. Analysis of this co-crystal structure, structures of all human isozymes in their autoinhibited state, and in-solution association studies showed that binding of Ca^2+^/CaM triggers large structural changes in the kinase domain as well as in the CaMKII regulatory domain that together lead to allosteric kinase activation. Furthermore, we also describe the structure of an oligomerization domain in its physiological, dodecameric state. Based on the comparison of this large body of structural information and biochemical characterization we propose a model that explains the substrate recognition leading to Ca^2+^/CaM-dependent allosteric activation of human CaMKIIs.

## Results

### Structures of Autoinhibited Human CaMKII Isozymes

**Figure 1:**
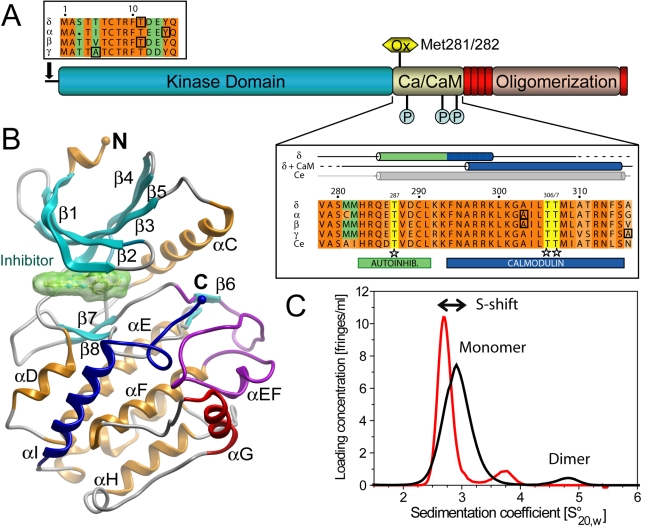
Structural features of CaMKII and dimerization of the kinase domain. A) Domain organization of CaMKII. The catalytic, regulatory and association domains are labelled, and predicted unstructured regions are shown in red. Sites of regulatory phosphorylation and oxidation are indicated. N- and C-terminal boundaries of the crystallized catalytic domain constructs are highlighted by boxed-in residues within the insets. The C-terminal boundary of CaMKIIδ, which is C-terminal to the range depicted (S333) has not been included in the figure. The organization of the autoinhibitory and the Ca^2+^/CaM binding domain is shown in the boxed alignment below the cartoon. For comparison, the sequence of the *C. elegans* (Ce) orthologue has also been included in the alignment. Secondary structural elements observed in the autoinhibited CaMKIIδ, CaMKIIδ/Ca^2+^/CaM, and *C. elegans* structure are shown above the alignment. The regions encompassing the Ca^2+^/CaM binding and autoinhibitory domains are highlighted in blue and green, respectively, and the phosphorylation sites are highlighted in yellow. B) Structural overview of the autoinhibited CaMKIIδ kinase domain refined at 2.3 Å resolution. The inhibitor bound at the ATP site is shown in surface representation in light-green, the activation segment is highlighted in magenta and main secondary structural elements are labelled. C) AUC sedimentation velocity experiment showing self-association of CaMKIIδ in absence (black line) and presence (red line) of a substrate-competitive peptide. The shift in sedimentation coefficient upon peptide binding is indicated by an arrow.

To date, our understanding of the molecular mechanisms that define the CaMKII autoinhibited state are based on the structural model of the *C. elegans* CaMKII orthologue (CeCaMKII). This crystal structure shows an occluded substrate binding site, rearrangements in the ATP binding site that disturb co-factor binding and a remarkable dimeric assembly involving the inhibitory helix and the CaM binding motif (corresponding to residues K293-F313 in CaMKIIα) [18]. CeCaMKII and human CaMKIIα share 77% sequence identity. We were interested in determining whether regulatory mechanisms suggested based on the crystal structure of CeCaMKII would be conserved in human CaMKII isozymes. To address this, we determined the structures of all human CaMKII isozymes in their autoinhibited state. The structures were refined at resolutions ranging from 2.25 Å (CaMKIIγ) to 2.4 Å (CaMKIIβ). Details of the diffraction data statistics and refinement have been summarized in Table S1. Importantly, whereas the crystallized constructs of the α and β isozymes contained the catalytic domain and the inhibitory region but only a part of the Ca^2+^/CaM binding motif, the constructs of both CaMKIIγ and CaMKIIδ additionally contained the entire regulatory region as well as a part of the unstructured linker to the association domain. The boundaries used for the crystallized proteins are shown in the boxed sequence inserts in Figure 1A and are indicated in the sequence alignment in Figure S1. As expected, based on the high sequence homology, all structures exhibited a high degree of structural similarity. The activation segments were all well-ordered and helix αC was correctly positioned for catalysis as indicated by formation of the conserved salt bridge between E60 located in αC and lysine K41, which is a hallmark of the active kinase conformation [30] (Figure S2).

Similar to the *C. elegans* orthologue, the substrate binding site is blocked by the regulatory domain in all human CaMKIIs (Figure 1B). However, dimeric association as in CeCaMKII, i.e., mediated by the regulatory domain, was not observed for any of the autoinhibited human isozymes. As mentioned above, the C termini of the crystallized γ and δ isozymes extended well beyond the Ca^2+^/CaM binding site (in CaMKIIδ, including the linker region up to S333). However, the autoinhibitory helices in all isozymes were structured only up to residue 302. Different crystal forms were observed, containing one (CaMKIIγ), two (CaMKIIα and CaMKIIδ), or four molecules (CaMKIIβ) in the asymmetric unit. However, no indication of a conserved dimer interface was evident within the crystals with packing contact regions typically involving small and diverse surface areas. However, evidence that dimerization occurs in solution was found using analytical ultracentrifugation (AUC) sedimentation velocity experiments for all CaMKII catalytic domains (exemplified by CaMKIIδ in Figure 1C), thus supporting studies that identified inactive CaMKII as paired (dimers) in cells [19]. The affinities, as estimated from the proportions between the areas of the peaks for monomeric and dimeric species, were weak (*K*_D_ of 200–600 µM), but due to the high effective concentrations of catalytic domains in the context of the holoenzyme, the observed interactions are likely to be biologically relevant (Table S2). Similar association constants were observed for all human CaMKIIs independent of the construct length, suggesting that dimerization is not mediated by the regulatory domain in human CaMKIIs. To test this hypothesis, we repeated the AUC experiments in the presence of an isolated regulatory domain peptide that spans the autoinhibitory region as well as the Ca^2+^/CaM binding site (CaMKIIδ residues 282–310). Interestingly, no change in the proportion between the peaks was observed (red trace in Figure 1C), suggesting that binding of the peptide did not interfere with dimerization in human CaMKIIs. Binding of the peptide led to a significant shift towards smaller sedimentation coefficients, suggesting that interaction with the peptide induces a conformational change that leads to increased friction. Based on these data, it is tempting to speculate that binding of the peptide displaces and subsequently causes unfolding of the inhibitory helix and the Ca^2+^/CaM binding site, as observed in the structure of the Ca^2+^/CaM complex.

### Structural Reorganization of the Catalytic and Inhibitory Domains Induced by Ca^2+^/CaM Binding

**Figure 2:**
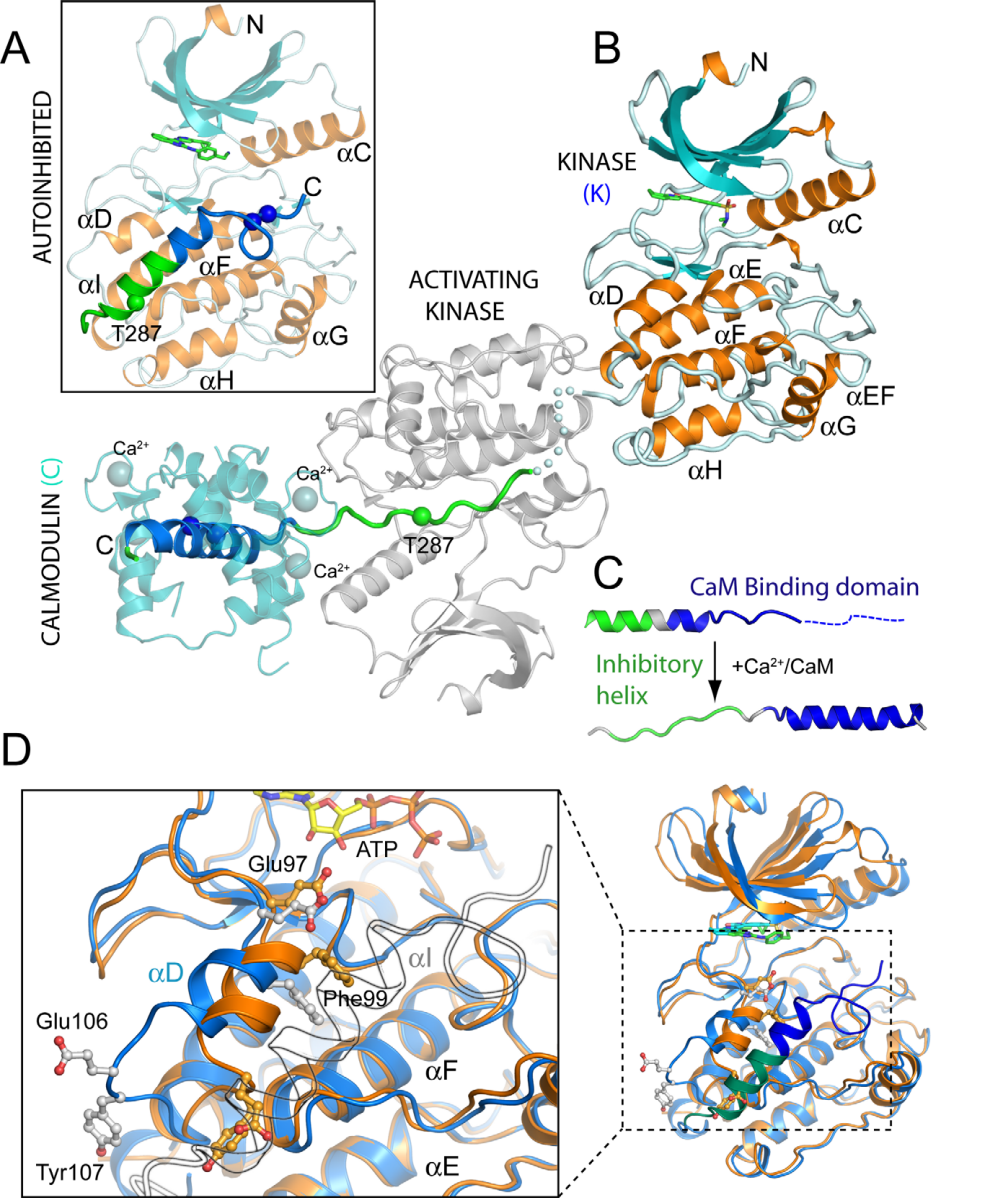
Structure of the CaMKIIδ/Ca^2+^/CaM complex. A) Ribbon diagram of the autoinhibited CaMKIIδ kinase featuring the regulatory domain blocking the substrate binding site. Regulatory phosphorylation sites (T287, T306, T307) are marked by spheres. The region encompassing the autoinhibitory sequence and the Ca^2+^/CaM binding domain are colored green and blue respectively throughout. Helices are coloured in orange, beta sheets in light-blue, and the ATP-competitive inhibitor is shown in stick representation. B) Ribbon diagram of the CaMKIIδ/Ca^2+^/CaM structure showing the regulatory region interacting with Ca^2+^/CaM (colored in cyan) and a symmetry-related *trans*-phosphorylating ‘activating’ catalytic domain (colored in gray). The kinase domain is shown in the same orientation as in A. For clarity, the Ca^2+^/CaM associated with the *trans*-phosphorylating kinase has been omitted in the figure. The phosphorylation site T287 is labeled and highlighted with a sphere. C) Structural rearrangement of the autoregulatory domain upon binding of Ca^2+^/CaM. The inhibitory helix and the Ca^2+^/CaM binding motif are colored in green and blue, respectively, similar to the other subfigures D) Structural rearrangements in the catalytic domain upon binding of Ca^2+^/CaM, shown by superimposing the structures of the catalytic domain in its autoinhibited (light blue) and active (orange) state. The right panel shows an overview and on the left a detailed view of the observed conformational changes in the lower kinase lobe is shown. For clarity, the autoinhibitory helix (αI) and coil regions are rendered transparent and outlined in black. Residues discussed within the text are labeled.

We were interested in exploring the structural consequences of Ca^2+^/CaM binding on CaMKII and determined the structure of the CaMKIIδ/Ca^2+^/CaM complex (Figure 2B). The structure of the complex comprised the catalytic and the regulatory domain of CaMKII (residues 1–333) and full length human calmodulin, and was refined at 1.9 Å resolution to an R/R_free_ of 16.1 and 19.9%, respectively (Table S1). In the complex, the regulatory region no longer interacted with its corresponding catalytic domain (Figure 2B). Instead, the conformation of the inhibitory region adopted an extended conformation, in sharp contrast to its helical secondary structure in autoinhibited CaMKII (Figure 2A). The observed extended conformation allowed interaction between the inhibitory region and the substrate binding site of an adjacent catalytic domain. Most notably, T287 was aligned in a position suitable for phosphoryl transfer (Figure 2B). Thus, the structure effectively “captures” CaMKIIδ in the process of transphosphorylation by a neighboring kinase molecule. The Ca^2+^/CaM binding region exhibits equally significant structural changes compared to the autoinhibited kinase. Although this region displays either an extended or partially disordered conformation in all human autoinhibited CaMKIIs, it adopts an entirely helical secondary structure in the Ca^2+^/CaM complex (Figure 2C).

The CaMKIIδ/Ca^2+^/CaM co-crystal structure revealed that phosphorylation at T287 is not the only mechanism that prevents the regulatory region from rebinding to the lower kinase lobe. In the complex, helix αD blocked the access to the binding site for the inhibitory helix by a significant reorientation of this helix with respect to the autoinhibited kinase; E106 and Y107, for instance, are displaced by more than 10 Å from their position (Cα) in the autoinhibited kinase and block the binding groove for the inhibitory helix (Figure 2D). The movement of αD has another important consequence which results in structural changes within the kinase active site: E97, which is oriented away from the ATP binding site in autoinhibited CaMKIIs, was positioned in a conformation that enables coordination of the ATP co-factor in the CaMKIIδ/Ca^2+^/CaM complex. This transition has been proposed previously based on in silico molecular dynamics simulations using the autoinhibited CeCaMKII structure in which the regulatory region was deleted [18]. It is well known that the affinity of CaMKII for ATP is significantly reduced in the absence of Ca^2+^/CaM [29],[31],[32]–[34]. E97 is highly conserved in kinases and plays a major role in recruiting ATP by forming interactions with the sugar moiety [35]. Similar conformations involving altered orientations of E97 that lead to kinase inactivation have been observed in autoinhibited structures of CaMK1 [36] and twitchin [37], suggesting that reorientation of αD is a common regulatory mechanism in CaMKs. An animation that illustrates the conformational changes that take place during Ca^2+^/CaM-dependent CaMKII activation has been embedded in the enhanced version of the manuscript (Datapack S1).

### Substrate Recognition of the T287 Phosphorylation Site

**Figure 3:**
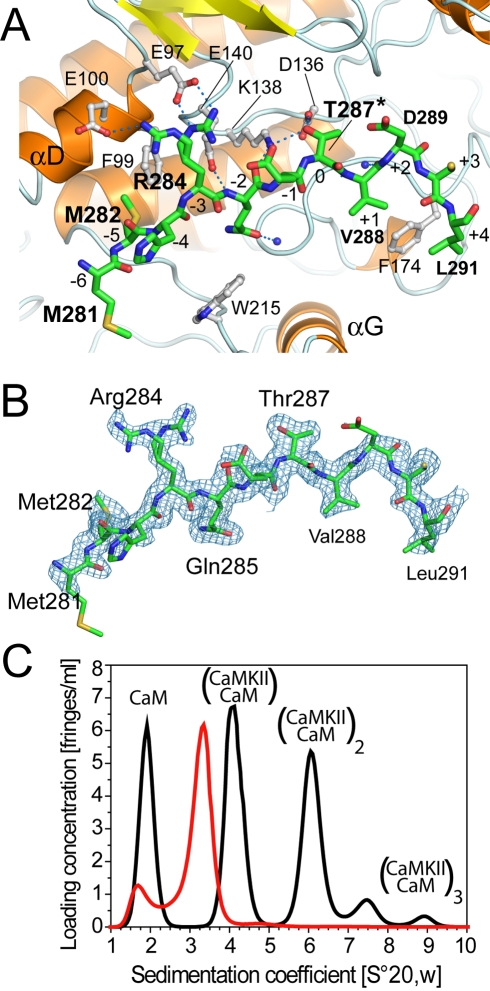
Details of the substrate-peptide interaction. A) Binding of the substrate sequence, including the regulatory site T287 (corresponding to T286 in the α isozyme), within the regulatory domain to the substrate binding pocket of an interacting catalytic domain. Substrate residues (green carbons) are labeled in bold and substrate positions are labeled with small Arabic numbers. The side chain of R284 forms hydrogen bonds with residues located in helix αD (E97/E100). Carbon atoms in catalytic domain residues are shown in light grey and residues are labeled in black. B) Electron density for the substrate peptide region. A region of the sigmaA-weighted electron density (2mFo-DFc) is shown contoured at 1σ superposed onto the final model. C) AUC velocity experiment showing CaMKIIδ/Ca^2+^/CaM association in the absence (black line) and presence (red line) of the substrate-competitive peptide encompassing the Ca^2+^/CaM binding region sequence. The assignment of the peaks to the oligomeric states (orders of association of CaMKII/Ca^2+^/CaM heterodimers) are indicated.

Transphosphorylation of T287 (T286 in CaMKIIα) by a catalytic domain present in the same holoenzyme has been described as the molecular switch that leads to constitutive and Ca^2+^/CaM-independent CaMKII activity [4],[21],[22]. The sequence flanking T287 represents a typical CaMK consensus substrate site [38]. The structure of the CaMKIIδ/Ca^2+^/CaM complex revealed how the regulatory region flanking T287 is recognized as a substrate: The arginine residue in position −3 (R284)—a hallmark of CaMK substrate recognition—exhibits two conformations and forms multiple polar interactions with the αD residues E100 and E97 (Figure 3A). The conformations of residues interacting within the substrate binding site were well defined in the electron density (Figure 3B). The structure of this substrate complex is similar to a CeCaMKII/peptide complex published recently [20].

We used analytical ultracentrifugation (AUC) to determine whether the CaMKII/Ca^2+^/CaM heterodimer forms in solution and to estimate affinities for the T287-substrate interaction. In agreement with our structural data, sedimentation velocity experiments measured on the CaMKIIδ/Ca^2+^/CaM complex revealed apparent molecular weights that correspond well to the calculated mass of the complex. The experiments also revealed the presence of oligomers containing two or more copies of the CaMKII/Ca^2+^/CaM heterodimer (Figure 3C). We estimated dissociation constants (*K_D_*) of 50 µM and 120 µM, for this self-association for the δ and α isozymes, respectively (Table S2). To further investigate whether the substrate binding pocket was indeed the interacting interface, we performed sedimentation velocity experiments in the presence of a substrate-competitive peptide. When a peptide derived from CaMKIIδ residues 282–310 was included, we observed only free Ca^2+^/CaM and the CaMKIIδ/Ca^2+^/CaM heterodimer in solution, but no higher association species. This experiment confirmed that the observed association is mediated by the substrate binding pocket recapitulating what was observed in the crystal structure of this complex.

### Recognition of the CaMKIIδ T307 Autophosphorylation Site

**Figure 4:**
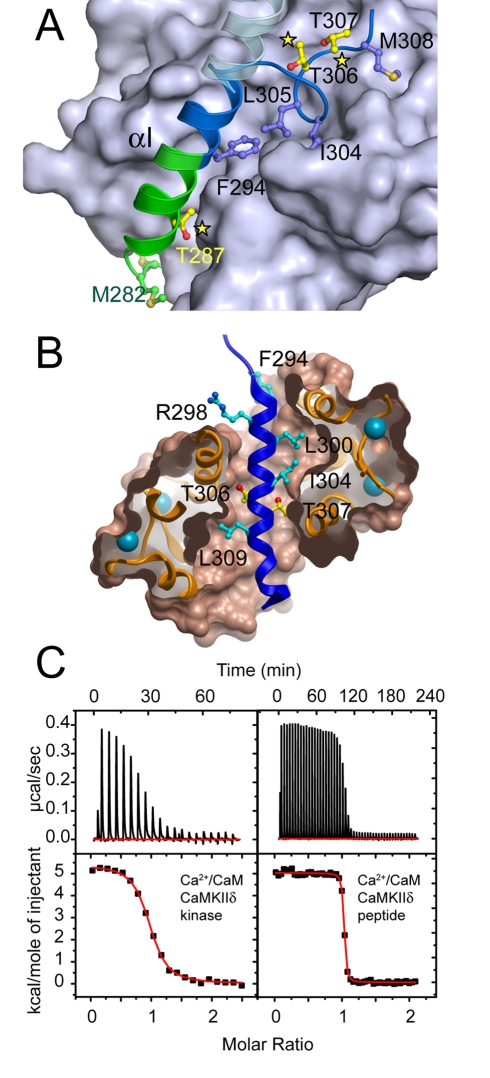
Recognition of the T307 autophosphorylation site and Ca^2+^/CaM binding. A) Substrate interaction of T307 in autoinhibited CaMKIIδ. The coloring of the inhibitory helix and the Ca^2+^/CaM-binding domain is similar to Figure 2 and important residues discussed in the text are annotated. Regulatory phosphorylation sites are highlighted by a yellow star. B) Details of the interaction between the Ca^2+^/CaM binding site (blue helix) and Ca^2+^/CaM, shown as ribbon with semitransparent binding surfaces. Residues located in the interface are labeled. C) Isothermal titration calorimetry (ITC) showing binding of Ca^2+^/CaM to CaMKIIδ (left panel) and the isolated Ca^2+^/CaM binding domain (right panel). The figure shows raw injection heats (upper panel) and a binding isotherm of normalized integrated binding enthalpies (lower panel). Experiments were carried out in 20 mM HEPES pH 7.5, containing 150 mM NaCl, 5 mM DTT and 1 mM CaCl_2_.

In inactive synapses, slow autophosphorylation at T306 (T307 in CaMKIIδ) accumulates a CaMKII pool of subunits that cannot be activated by Ca^2+^/CaM and requires phosphatase activity for reactivation [39],[40]. The sequence flanking T307 represents a site that is incompatible with CaMKII consensus substrate requirements, raising the question as to how this regulatory phosphorylation site would be recognized as a kinase substrate. In the CaMKIIδ structure, T307 was bound to the substrate binding site and oriented in an identical fashion to that observed for T287 in the transphosphorylating complex. The T307-containing region adopts an unusual turn conformation not previously seen in kinase substrate complexes. This unusual binding mode was stabilized by hydrophobic interactions of the conserved residues I304 and L305 that bound inside a deep cavity formed by residues located in the P1 loop and in helix αG (Figure 4A). This hydrophobic anchor allows recognition of this non-consensus substrate site, thus providing insight into how T307 is recognized as a *cis*-autophosphorylation site.

### Binding of Ca^2+^/CaM

Ca^2+^/CaM has the ability to bind to a large number of distinct proteins by adjusting the relative orientation of its EF hands [41]–[43]. Residues in the interface were well-defined and the recognition motif bound tightly within the central cavity of the Ca^2+^/CaM structure while no stable contacts were made with the kinase domain. The Ca^2+^/CaM interaction involved the CaMKII residues 296–316. Comparison with the autoinhibited structures of human CaMKII isozymes revealed that a helical secondary structure was induced upon Ca^2+^/CaM binding for the region C-terminal to L300. The interactions between CaMKII and Ca^2+^/CaM were largely mediated by hydrophobic contacts in the interface of Ca^2+^/CaM and the CaMKII binding helix. Phosphorylation of T306 in CaMKIIα (T307 in other CaMKII isoforms), located within the Ca^2+^/CaM binding domain, has been shown to inhibit Ca^2+^/CaM binding [29],[25]. Moreover, Ca^2+^/CaM binding to CaMKII with unphosphorylated T306 effectively prevents phosphorylation of this residue [26]. These data are in agreement with our co-crystal structure that showed that both threonine residues (T306/T307) were deeply buried within the Ca^2+^/CaM complex (Figure 4B).

We used isothermal titration calorimetry (ITC) to compare the affinities of Ca^2+^/CaM to autoinhibited CaMKII catalytic domains with those of the isolated regulatory domain and catalytic domains of CaMKI and CaMKIV: Human CaMKII kinase domains bound Ca^2+^/CaM with affinities between 1.6 and 3.4×10^6^ Mol^−1^ (*K_D_*: 0.6–0.3 µM) (Figure 4C, Table 1). The determined binding affinities were in agreement with affinities determined for the full-length enzyme [33]. Ca^2+^/CaM bound to CaMKI and CaMKIV with >20-times higher affinity. Interestingly, these values compared well with the affinity of the isolated Ca^2+^/CaM binding domain (CaMKIIδ residues 296–315) (97.3×10^6^ Mol^−1^; *K_D_*: 10.2nM). Comparison with the affinity of the isolated regulatory domains suggests that an energy barrier of ∼2.3 kcal/mol is associated with the release of the inhibitory helix and the Ca^2+^/CaM trapping mechanism [33]. A unique feature of the CaMKII interaction is the unfavorable (positive) binding enthalpy. The observation that this thermodynamic fingerprint of the binding to Ca^2+^/CaM is shared by all human CaMKIIs, but neither by CaMKI nor CaMKIV, underlines the mechanistic differences between these classes of CaMK.

**Table 1:** Isothermal titration calorimetry data of the interaction of Ca^2+^/CaM with CaMKs.

	Concentration (mM)**	*K*_B_×10^6^ (M^−1^)*	Δ*H* ^obs^ (kcal/mol)*	TΔS (kcal/mol)	Δ*G* (kcal/mol)	N
CaMKIIδ - Ca^2+^/CaM	0.266/0.012	3.7±0.3	+5.4±0.05	13.8	−8.4	0.933
CaMKIIδ - pep- Ca^2+^/CaM	0.532/0.038	97.3±10	+4.4±0.03	14.7	−10.3	1.020
CaMKIIα - Ca^2+^/CaM	0.150/0.015	1.7±0.08	+12.1±0.13	20.2	−8.0	0.863
CaMKIIβ - Ca^2+^/CaM	0.112/0.010	2.9±0.2	+8.9±0.08	17.3	−8.4	0.922
CaMKIIγ - Ca^2+^/CaM	0.110/0.010	1.9±0.15	+8.6±0.1	16.7	−8.1	0.981
CaMKI - Ca^2+^/CaM	0.150/0.015	187.0±84	−5.8±0.07	4.9	−10.7	1.030
CaMKI - Ca^2+^/CaM	0.248/0.024	78.8±21	−5.1±0.04	5.1	−10.2	0.930
CaMKIV - Ca^2+^/CaM	0.156/0.088	108.0±19	−10.2±0.05	0.14	−10.3	0.830

*Deviations from a non-linear least squares fit. Each data set was determined from a single titration experiment. The error was estimated from a repeated experiment (performed for CaMKI, labeled with ^§^) employing re-purified CaMKI and re-purified Ca^2+^/CaM. We found that binding constants had an error of approximately two-fold with, however, similar binding enthalpy and stoichiometry.

**Values represent the concentration of the titrant (Ca^2+^/CaM) and the protein (the kinase domain) in the calorimeter. For the experiment on the isolated regulatory domain (CaMKIIδ pep: peptide, ARRKLGAILTTMLATRNF corresponding to Ca^2+^/CaM binding domain residues 296–315 CaMKIIδ), the peptide was titrated into a solution containing Ca^2+^/CaM.

N: Stoichiometry of the interaction determined in the experiment.

### Oligomeric Assembly of CaMKII

**Figure 5:**
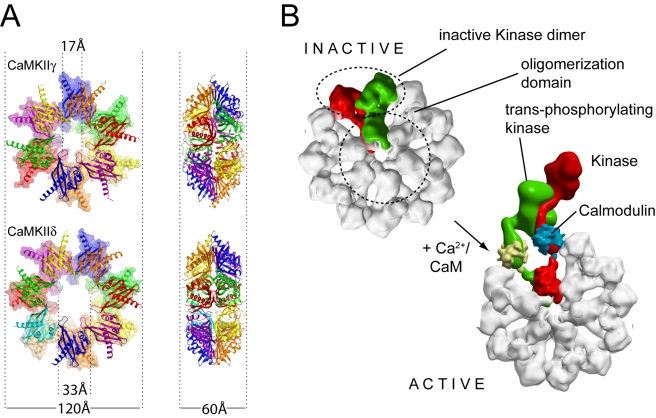
Oligomerization domains of human CaMKIIδ and CaMKIIγ. A) Comparison between the structures of the dodecameric (CaMKIIγ) (upper) and tetradecameric (CaMKIIδ) (lower) arrangements. The rings are viewed looking face-on (left) and side-on (right) B) Model of the autoactivation of full-length CaMKII. For clarity, only one CaMKII dimer is highlighted (protomers shown in green and red, respectively). The model was constructed by manual docking of the kinase domain and the CaMKII/Ca^2+^/CaM complex to the experimental structure of the dodecameric oligomerization domain.

Oligomerization into large ring-like structures is a unique feature of CaMKIIs and the oligomeric state is crucial for rapid autophosphorylation in *trans* by catalytic domains present in the same oligomer [44]. The discrepancy between the dodecameric (12-mer) structures determined by electron microscopy [13],[14] and the tetradecameric (14-mer) assembly revealed by the crystal structure of the isolated oligomerization domain of the *C. elegans* orthologue [15] prompted us to crystallize the oligomerizaton domains of human CaMKII isozymes. Here we present the oligomerization domains of the human γ and δ isozymes that were refined at 2.7 and 2.8 Å resolution, respectively. The quality of the final model was substantially improved by averaging the electron density maps using non-crystallographic symmetry. While isolated domains of the δ isozyme were tetradecameric, the CaMKIIγ oligomerization domain crystallized in its dodecameric state, thus providing a model for the oligomerization state observed in full-length CaMKIIs. The oligomerization domain formed a hexameric structure with a diameter of 120 Å and a height of 60 Å surrounding a central cavity of only 17 Å (Figure 5A). Thus, the main consequence of the insertion of an additional subunit per ring in the tetradecameric assembly is a considerable widening of the central cavity, to about 33 Å.

Each oligomerization domain in the ring also contains a deep cavity that has been suggested to represent a peptide binding site, based on structural homology with peptide-binding domains present in nuclear transport factors and scytalone dehydratase [15]. In support of this hypothesis is the fact that a glycine or an acetate molecule, respectively, originating from the crystallization solution were partially occupying this putative binding site in the structures of the CaMKIIγ and CaMKIIδ oligomerization domains.

We used the structures of the dodecameric oligomerization domain, the CaMKIIδ/Ca^2+^/CaM complex and the structures of the inactive catalytic domains, to construct models for the full-length enzyme (Figure 5B). In our model of the autoinhibited protein, catalytic domains located in both hexameric rings associate. The pairing was chosen arbitrarily whilst based on the orientation of the helices in the dodecameric oligomerization domain. Binding of Ca^2+^/CaM triggers unfolding of the inhibitory helix and releases the kinase domain. This structural reorganization allows T287 to bind to an adjacent kinase domain, leading to autophosphorylation and restructuring of the kinase domain to form a conformation with high affinity for ATP due to reorientation of αD. Once T287 has been phosphorylated, the kinase domain is released fully active and independent of Ca^2+^/CaM, and with its substrate binding site accessible.

## Discussion

**Figure 6:**
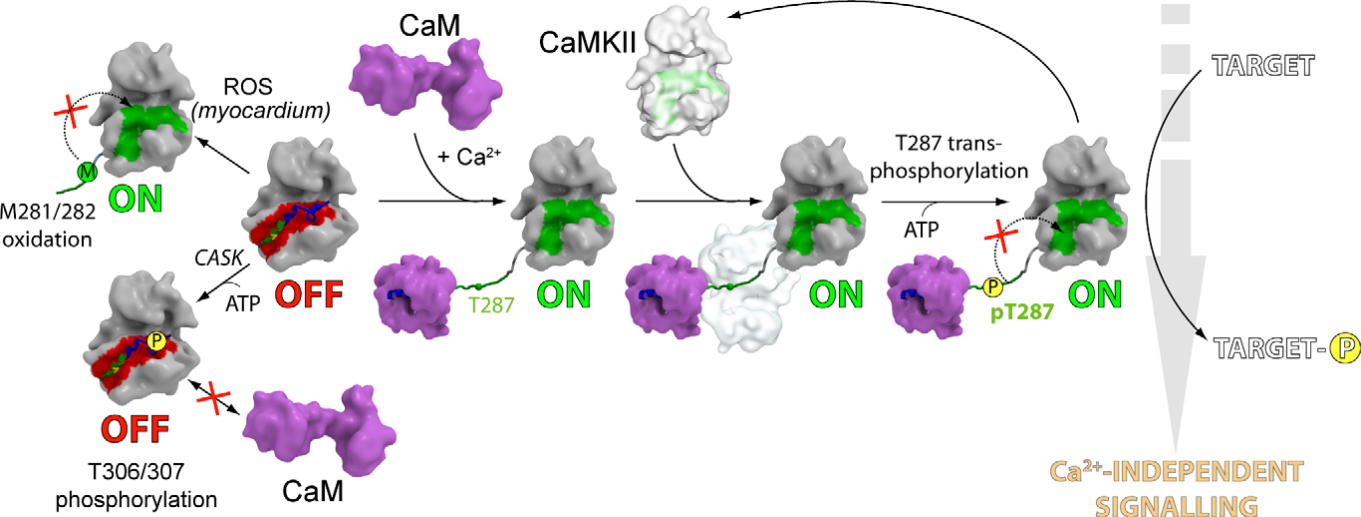
Putative mechanisms of CaMKII activation and inactivation. In autoinhibited CaMKII (second from the left), the inactivating C-terminal helix is bound to the substrate binding site (shown as a red surface). Ca^2+^/CaM-independent mechanisms lead to an active state (“ON” state; to the top left) by methionine oxidation, or to an inactive state (“OFF” state; bottom left) by CASK-mediated T306/T307 phosphorylation. Ca^2+^/CaM-dependent activation (to the right) is achieved through structural rearrangement of the inhibitory helix caused by Ca^2+^/CaM binding and subsequent autophosphorylation of T287. Even when calmodulin is released from the complex following a drop in the Ca^2+^ level, re-binding of the inhibitory helix is blocked (marked as a red cross) due to pT287 and a structural change in the lower kinase lobe that closes the binding site for the helix, restructures the substrate binding site (depicted as a green surface), and aligns E97 in the active site for ATP binding. As a consequence, the active state of the kinase is long-lasting and signaling pathways that lead to changes in gene transcription are activated.

Four regulatory features distinguish the regulation of CaMKII isozymes from other CaMKs. Firstly, CaMKIIs form large oligomers bringing catalytic domains into close proximity to facilitate rapid autoactivation. Secondly, autophosphorylation of T287 generates Ca^2+^/CaM-independent sustained activity. Thirdly, phosphorylation of T287 increases the affinity of CaMKII for Ca^2+^/CaM by more than 10,000-fold, an effect known as CaM-trapping [45]. Fourthly, phosphorylation of T306/T307 leads to prevention of CaMKII activation, due to an interference with Ca^2+^/CaM binding. Comparison of the active CaMKII with its inactive autoinhibited structures provides a structural model for the unique switch-like mechanism of CaMKII autoactivation, as well as for its inactivation by autophosphorylation of T307. The different molecular mechanisms of CaMKII activation and inactivation are depicted in Figure 6, and we discuss here the structural background of these regulatory events in the light of the determined structures.

Autophosphorylation of T307 and its neighbor T306 prevents binding of Ca^2+^/CaM, resulting in inhibitory autophosphorylation [44]. Moreover, knock-in mice expressing mutants of CaMKIIα incapable of phosphorylation of T306 (e.g. T306A, corresponding to T307 in CaMKIIδ) identified this residue as an important site regulating synaptic plasticity [46]. However, if T287 is phosphorylated prior to T307, Ca^2+^/CaM-independent activity is induced, whereas prior phosphorylation of T307 prevents activation and autophosphorylation of T287. This illustrates that the sequence of autophosphorylation events is a critical component of CaMKII regulation [47]. In agreement with the observation that ATP binding is impaired in autoinhibited CaMKII, we observed only slow autophosphorylation activity of CaMKIIδ in vitro minimizing premature phosphorylation of T307 (unpublished data). This phosphorylation is stimulated by interaction with CASK [25],[26]. Based on our model of inactive CaMKIIs, it is likely that binding of CASK leads to an allosteric rearrangement similar to the one observed upon release of the inhibitory helix that results in CaMKII activation and autophosphorylation of T306/T307 by the described non-canonical binding mode of the non-consensus substrate site at T307 in inactive CaMKIIδ.

A recent report showed that Angiotensin II-induced oxidation of the methionines M281/M282 leads to sustained activity of CaMKII in the absence of Ca^2+^/CaM, inducing apoptosis in cardiomyocytes [48]. The structure of inactive CaMKII suggests that oxidation of M282 would lead to steric clashes with the lower lobe, resulting in destabilization of the autoinhibited state and Ca^2+^/CaM-independent activation. In the CaMKIIδ/Ca^2+^/CaM complex, M282 binds into a tight hydrophobic pocket in the substrate binding site, suggesting that oxidation of this residue also interferes with substrate binding and autophosphorylation of T287 (see Figure 3A and 4A). Taken together, our structural data suggest that Met oxidation interferes with both the CaMKII inactive state and autophosphorylation of T287, thus supporting a role of M282 oxidation in CaMKII regulation [48]. The key discoveries of this study were conformational changes that take place upon binding of Ca^2+^/CaM, resulting in activation of the kinase. These structural rearrangements were identified by detailed comparison between the structures of all autoinhibited CaMKIIs and the CaMKII/Ca^2+^/CaM co-crystal structure. Interestingly, CaM did not interact with any other region of the catalytic domain of CaMKII outside the helical recognition motif. The absence of a specific docking site for Ca^2+^/CaM on the catalytic domain proper presumably allows this versatile modulator to interact with a large number of highly diverse proteins by inducing structural rearrangements in its target enzymes. Recently, the structure of a complex of Ca^2+^/CaM with DAPK1 (Death Associated Protein Kinase 1) revealed multiple interactions of Ca^2+^/CaM with the upper and lower kinase lobe and binding of Ca^2+^/CaM in an extended conformation [49]. In addition, the substrate binding site in the DAPK1/Ca^2+^/CaM complex was occluded by Ca^2+^/CaM, suggesting that a conformational change would need to take place to fully activate DAPK1. CaMKIIs are only distantly related to DAPKs, and it seems that the interaction with Ca^2+^/CaM and the mechanism of activation of these two CaMKs are fundamentally different.

All four CaMKII isozymes were crystallized with ATP-competitive inhibitors that are relatively non-selective. However, their binding modes (see Figure S3) may nonetheless provide valuable chemical starting points for structure-based design of selective and potent inhibitors for the treatment of diseases. CaMKIIs have been implicated in heart failure [50], arthritis [51], and certain types of cancer [52]–[54]. The detailed comparison of the large body of structural information presented here provides the first insight into how an intact CaMKII catalytic/regulatory domain interacts with Ca^2+^/CaM. However, issues such as how catalytic domains pair together or how activation resulting from *trans*-phosphorylation is propagated in the holoenzyme would be best addressed by structures of full length CaMKII, an effort that is ongoing in our laboratory.

## Material and Methods

### Protein Expression and Purification

Expression constructs comprised the following residues of human CaMKs: CaMKIδ 1–333, CaMKIIα 13–301, CaMKIIβ 11–302 and 358–498, CaMKIIδ11–335 and 334–475, CaMKIIγ 5–317 and 387–527, CaMKIV 15–340, and calmodulin 1–152 were cloned into the T7 expression vector pNIC28-Bsa4 by ligation-independent cloning. Proteins were expressed in *Escherichia coli* BL21(DE3)R3 as fusions to a Tobacco Etch Virus (TEV)–cleavable N-terminal His_6_ affinity tag. Cells were re-suspended in lysis buffer (50 mM HEPES pH 7.5 at 25°C, 0.3 M NaCl, 20 mM imidazole) in the presence of a protease inhibitor mix (Complete, EDTA-free Protease Inhibitor Cocktail, Roche Diagnostics Ltd.) and lysed using an EmulsiFlex-C5 high pressure homogenizer (Avestin) or, alternatively, by sonication at 4°C. The lysate was bound to a Ni-NTA column, extensively washed (50 mM HEPES pH 7.5, 300 mM NaCl, 20 mM imidazole) and eluted using the same buffer containing 200 mM imidazole. Proteins were dephosphorylated in vitro with the addition of 50 mM MnCl_2_ and λ-phosphatase overnight at 4°C. The eluted protein was pooled, concentrated and applied to either a Superdex 75 or 200 16/60 HiLoad gel filtration column equilibrated in 50 mM HEPES, pH 7.5, 300 mM NaCl, 10 mM dithiothreitol (DTT). Additional purification by ion-exchange chromatography (HiTrap Q in 50 mM Tris pH 8.8, using 0.1 to 0.7 M NaCl gradients) was used where purification was insufficient. Purity was monitored by SDS-polyacrylamide gel electrophoresis and final samples were concentrated to 10–15 mg/ml. Dephosphorylation.of CaMKIIγ was inefficient, preventing TEV-cleavage due to phosphorylation of the TEV recognition motif. The alpha and gamma isozymes were crystallized in fusion with the His_6_-TEV tag.

### Crystallization and X-Ray Data Collection

Crystals were obtained by the sitting drop vapour diffusion method at 4°C using conditions included in Table S1. Inhibitors used for co-crystallization were added to the protein solutions prior to crystallization at a final concentration of 1 mM. Diffraction data were collected on beam-line X10SA at the Swiss Light Source (SLS, Paul-Scherrer Institute, Villigen, Switzerland) from crystals flash-frozen in liquid nitrogen.

### Data Processing, Molecular Replacement and Refinement

Data were indexed and integrated using MOSFLM [55] and were scaled using SCALA [56]. Structures were phased by molecular replacement using PHASER [57] with the coordinates of either the *C. elegans* model of inactive catalytic domain [18] or the refined CaMKIIγ, respectively, as search models. Refinement was carried out using REFMAC5 [58] employing appropriate geometric and non-crystallographic restraints. The models and structure factors have been deposited with PDB accession codes listed in Table S1.

### Analytical Ultracentrifugation

Sedimentation velocity experiments were carried out on an Optima XL-I Analytical Ultracentrifuge (Beckman Instruments, Palo Alto, CA) equipped with a Ti-50 rotor. Protein samples were studied at a various concentrations in 10 mM HEPES pH 7.5, containing 300 mM NaCl, 1 mM CaCl_2_ and 5 mM DTT at 4°C, employing a rotor speed of 50,000 rpm. Radial absorbance scans were collected in one-minute intervals using a double-sector cell. 300 µl aliquots were loaded into the sample channels of double-channel 12-mm centerpieces and 310 µl of buffer into the reference channels. Data were analyzed using SEDFIT [59],[60] to calculate c(s) distributions. SEDNTERP was used to normalize the obtained sedimentation coefficient values to the corresponding values in water at 20°C, 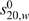. Translational frictional ratios 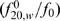 were calculated from the *s_20,w_* values, using:
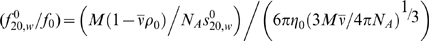
where *M* is the molecular weight, 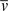 is the partial specific volume, *N_A_* is Avogadro's number and 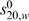 is the sedimentation coefficient corrected to the standard conditions of density, *ρ_0_*, and viscosity, *η_0_*, of water at 20.0°C, and extrapolated to infinite dilution. Sedimentation equilibrium experiments were performed at 4°C and at a number of protein concentrations. Dissociation constants, *K*_d_, were calculated, respectively, from the fitted apparent association constants, *K*_a,obs_, according to the equation
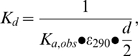
where d is the optical pathlength and ε_290_ the extinction coefficient at 290 nm.

### Isothermal Titration Calorimetry

ITC data were measured using a VP-ITC titration microcalorimeter from MicroCal, LLC (Northampton, MA). For all experiments the proteins used were dialyzed against 20 mM HEPES pH 7.5, containing 150 mM NaCl, 5 mM DTT and 1 mM CaCl_2_. Data were measured at 10°C by titrating Ca^2+^/CaM into CaMKII catalytic domains or peptides into Ca^2+^/CaM, respectively. The inhibitory peptide sequence used for titrations was ARRKLGAILTTMLATRNF, corresponding to Ca^2+^/CaM binding domain residues 296–315 in CaMKIIδ. Each experiment consisted of a first injection of 2 µl followed by 29 injections of 10 µl injected during 20 s and a spacing of 280 s between injections. Blank titrations (Ca^2+^/CaM into buffer) were subtracted from the titration data. Data were normalized and evaluated using ORIGIN with a single binding site model. Thermodynamic parameters were calculated using: Δ*G* = Δ*H*−TΔ*S* = −RTln*K*_B_, where Δ*G*, Δ*H* and Δ*S* are the changes in free energy, enthalpy and entropy of binding, respectively.

## Supporting Information

Datapack S1 **Standalone iSee datapack - contains the enhanced version of this article for use offline.** This file can be opened using free software available for download at http://www.molsoft.com/icm_browser.html.
(ICB)

Figure S1 **Sequence alignment of human CaMKIIs with the** ***C. elegans*** **(Ce) orthologue.** The regulatory and linker domains are boxed. The regulatory phosphorylation sites within the inhibitory domain are indicated by yellow stars. Catalytic/nucleotide-binding residues are highlighted by red stars. The secondary structure of CaMKIIδ is shown above the alignment (green). However, for the regulatory region, secondary structures are indicated for all kinases in the alignment including CaMKIIδ in the absence (IIδ-CaM) and presence (IIδ+CaM) of calmodulin. Dotted regions correspond to disordered regions that were not modelled. The numbering above the alignment corresponds to the sequence of CaMKIIδ. The start/end residues for each construct used in crystallization are boxed in each sequence. Alignment prepared using ALINE (Bond, C.S. and Schüttelkopf, A.W. (2009), Acta Cryst. D65, 510–512).
(2.91 MB DOC)

Figure S2 **Structural overview and active site features of autoinhibited human CaMKII isozymes.** A) Superimposition of the 4 human isozymes (CaMKIIα – yellow; CaMKIIβ – grey; CaMKIIδ – green; CaMKIIγ – orange). Structures have been superimposed using the lower lobe as a reference and differences in domain orientation of the upper lobe are evident. The tip of the P-loop is not ordered in the alpha and gamma isozymes. The main structural elements are labelled; the inhibitory helix has been highlighted in blue. B) Positioning of the helix αC in the active site of human CaMKII isozymes. Salt bridges between the conserved active site lysine (K41) and the αC glutamate (E60/61) were all between 2.7 Å and 2.8 Å, indicating an active conformation of this helix. The loop region linking the sheet β3 and αC as well as the αC N terminus showed a high degree of conformational variability, suggesting that these structural elements are quite flexible despite the constitutively active nature of the CaMKII kinase domain. C) CaMKII isozymes do not require activation segment phosphorylation for activity and the site typically phosphorylated in kinases (−11 residues from APE motif) is substituted by a highly conserved glycine residue (G173). In the absence of phosphorylation the conformational stability of the activation segment is increased by a hydrogen bond network formed by the catalytic loop R135, the backbone oxygen of L160, G173 and P172 as well as with the side chain oxygen of a highly conserved tyrosine (Y191) located in the loop linking the activation segment with helix αF. In addition, the tip of the activation segment is stabilized by a conserved cluster of hydrophobic residues (F172, I161, V164, A170) conserved in all CaMKII isozymes and most orthologues.
(2.55 MB DOC)

Figure S3 **Binding of ATP-competitive inhibitors to the four CaMKII isozymes.** The binding mode of each inhibitor is shown in the upper panel and the inhibitor structure in the lower panel.
(2.53 MB DOC)

Table S1 Structural refinement and data collection.
(0.07 MB DOC)

Table S2 Dissociation constants for the formation of dimers of CaMK catalytic domains and of “dimers of CaMKII Ca^2+^/CaM heterodimers” (CaMKII-CaM)_2_. Protein association was monitored using analytical ultracentrifugation sedimentation velocity experiments, in 10 mM HEPES buffer containing 300 mM NaCl, 1 mM CaCl_2_ and 5 mM DTT, at 4°C. Distributions of species were analyzed using SEDFIT and dissociation constants were estimated from the proportions between monomer/dimer and heterodimer/heterotetramer peaks, respectively, using *K*_D_ = c*(m)2/d, where c, concentration in molar; m, proportion of monomers; d, proportion of dimers.
(0.05 MB DOC)

Text S1 Instructions for installation and use of the required web plugin (to access the online enhanced version of this article).
(0.75 MB PDF)

## Abbreviations

Ca^2+^: calcium
CaM: calmodulin
CaMK: calmodulin-dependent kinase
DAPK1: Death Associated Protein Kinase 1
LTP: long-term potentiation
