# Supplementary figures and images for: Structure of the CaMKIIδ/Calmodulin Complex Reveals the Molecular Mechanism of CaMKII Kinase Activation
Source: PLoS Biol. 2010 Jul 27;8(7):e1000426. doi: 10.1371/journal.pbio.1000426 (PMC2910593; doi:10.1371/journal.pbio.1000426)

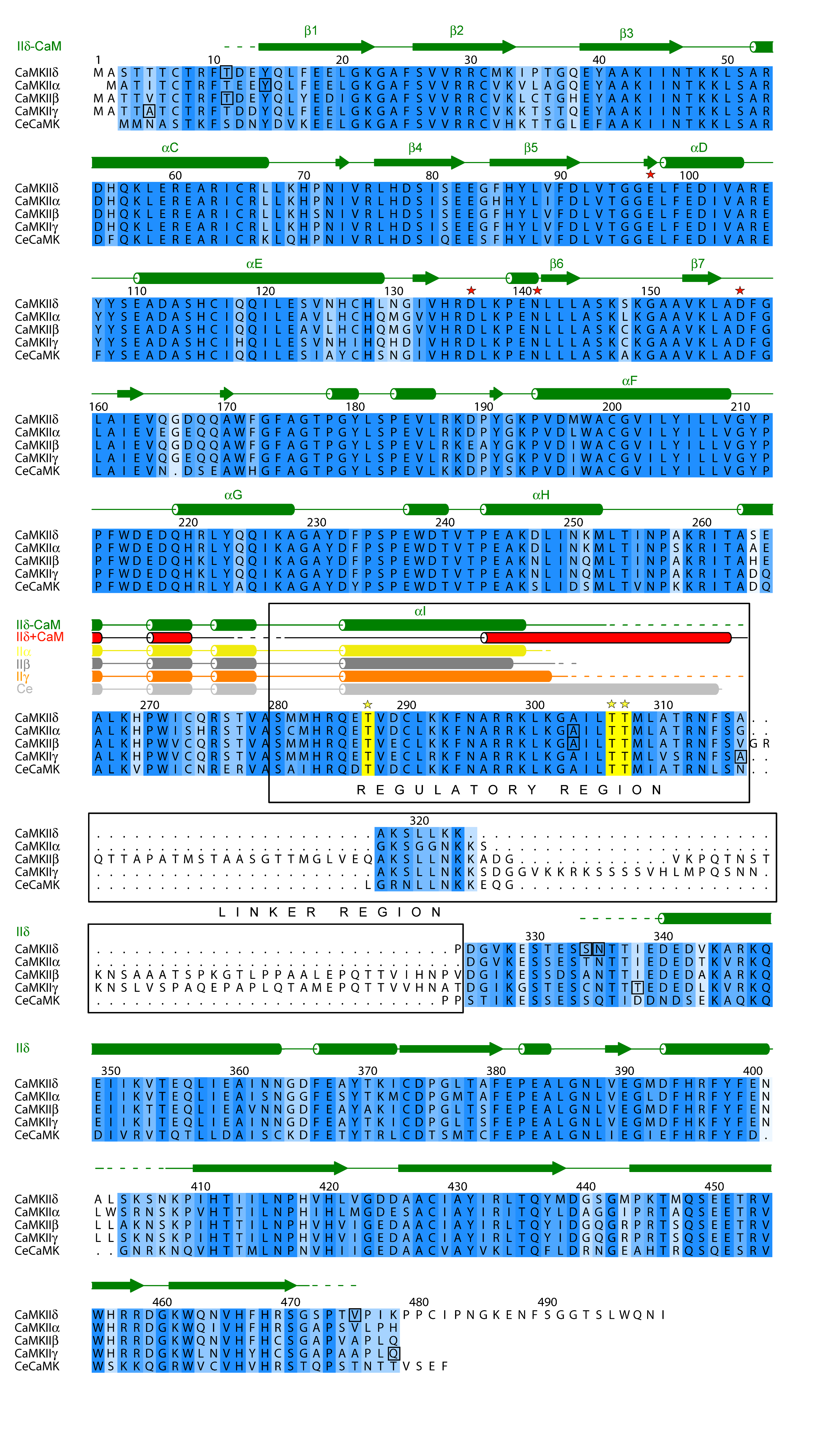

Supplement: Figure S1 — Sequence alignment of human CaMKIIs with the C. elegans (Ce) orthologue. The regulatory and linker domains are boxed. The regulatory phosphorylation sites within the inhibitory domain are indicated by yellow stars. Catalytic/nucleotide-binding residues are highlighted by red stars. The secondary structure of CaMKIIδ is shown above the alignment (green). However, for the regulatory region, secondary structures are indicated for all kinases in the alignment including CaMKIIδ in the absence (IIδ-CaM) and presence (IIδ+CaM) of calmodulin. Dotted regions correspond to disordered regions that were not modelled. The numbering above the alignment corresponds to the sequence of CaMKIIδ. The start/end residues for each construct used in crystallization are boxed in each sequence. Alignment prepared using ALINE (Bond, C.S. and Schüttelkopf, A.W. (2009), Acta Cryst. D65, 510–512). (2.91 MB DOC) [file pbio.1000426.s002.tif]

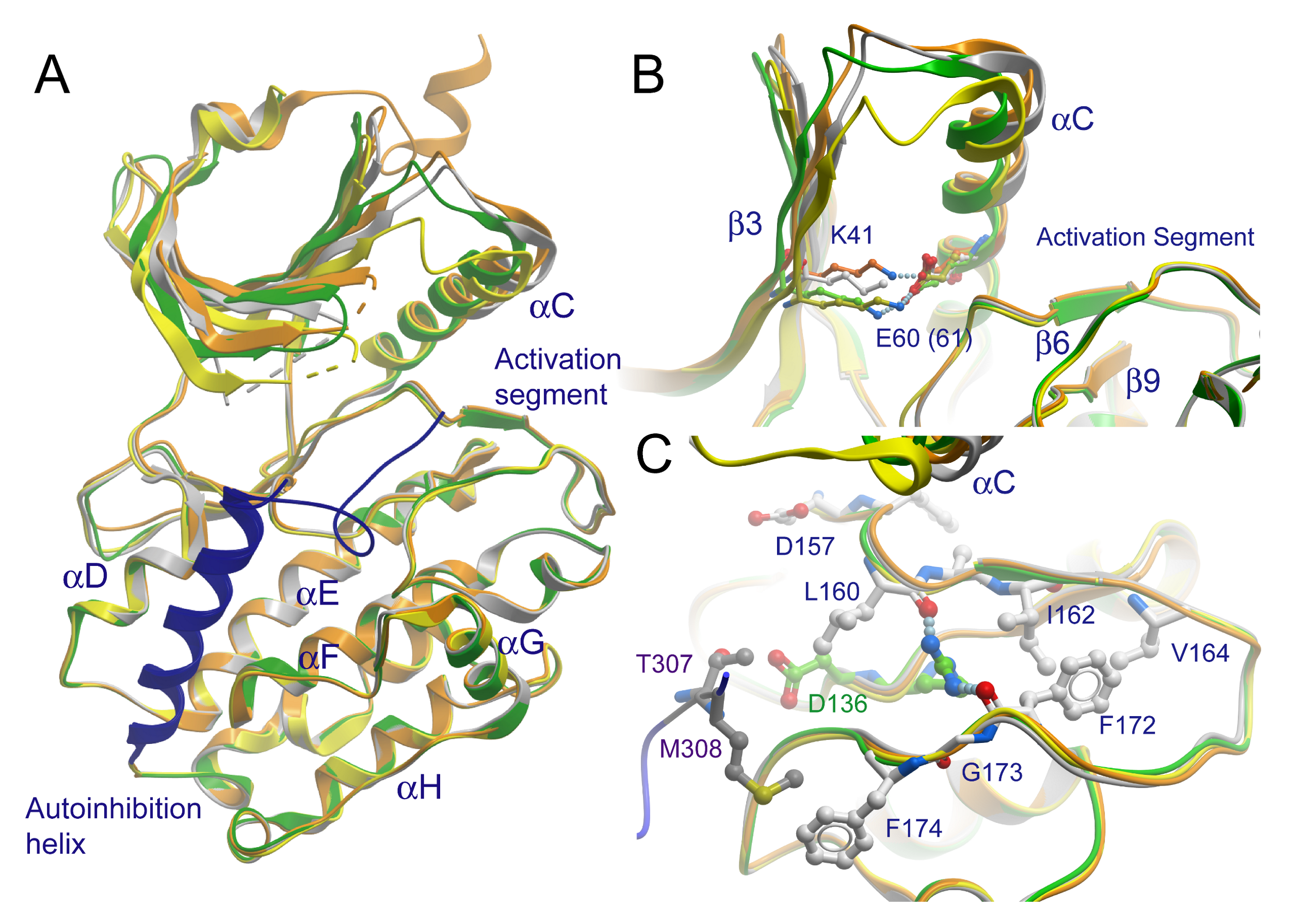

Supplement: Figure S2 — Structural overview and active site features of autoinhibited human CaMKII isozymes. A) Superimposition of the 4 human isozymes (CaMKIIα – yellow; CaMKIIβ – grey; CaMKIIδ – green; CaMKIIγ – orange). Structures have been superimposed using the lower lobe as a reference and differences in domain orientation of the upper lobe are evident. The tip of the P-loop is not ordered in the alpha and gamma isozymes. The main structural elements are labelled; the inhibitory helix has been highlighted in blue. B) Positioning of the helix αC in the active site of human CaMKII isozymes. Salt bridges between the conserved active site lysine (K41) and the αC glutamate (E60/61) were all between 2.7 Å and 2.8 Å, indicating an active conformation of this helix. The loop region linking the sheet β3 and αC as well as the αC N terminus showed a high degree of conformational variability, suggesting that these structural elements are quite flexible despite the constitutively active nature of the CaMKII kinase domain. C) CaMKII isozymes do not require activation segment phosphorylation for activity and the site typically phosphorylated in kinases (−11 residues from APE motif) is substituted by a highly conserved glycine residue (G173). In the absence of phosphorylation the conformational stability of the activation segment is increased by a hydrogen bond network formed by the catalytic loop R135, the backbone oxygen of L160, G173 and P172 as well as with the side chain oxygen of a highly conserved tyrosine (Y191) located in the loop linking the activation segment with helix αF. In addition, the tip of the activation segment is stabilized by a conserved cluster of hydrophobic residues (F172, I161, V164, A170) conserved in all CaMKII isozymes and most orthologues. (2.55 MB DOC) [file pbio.1000426.s003.tif]

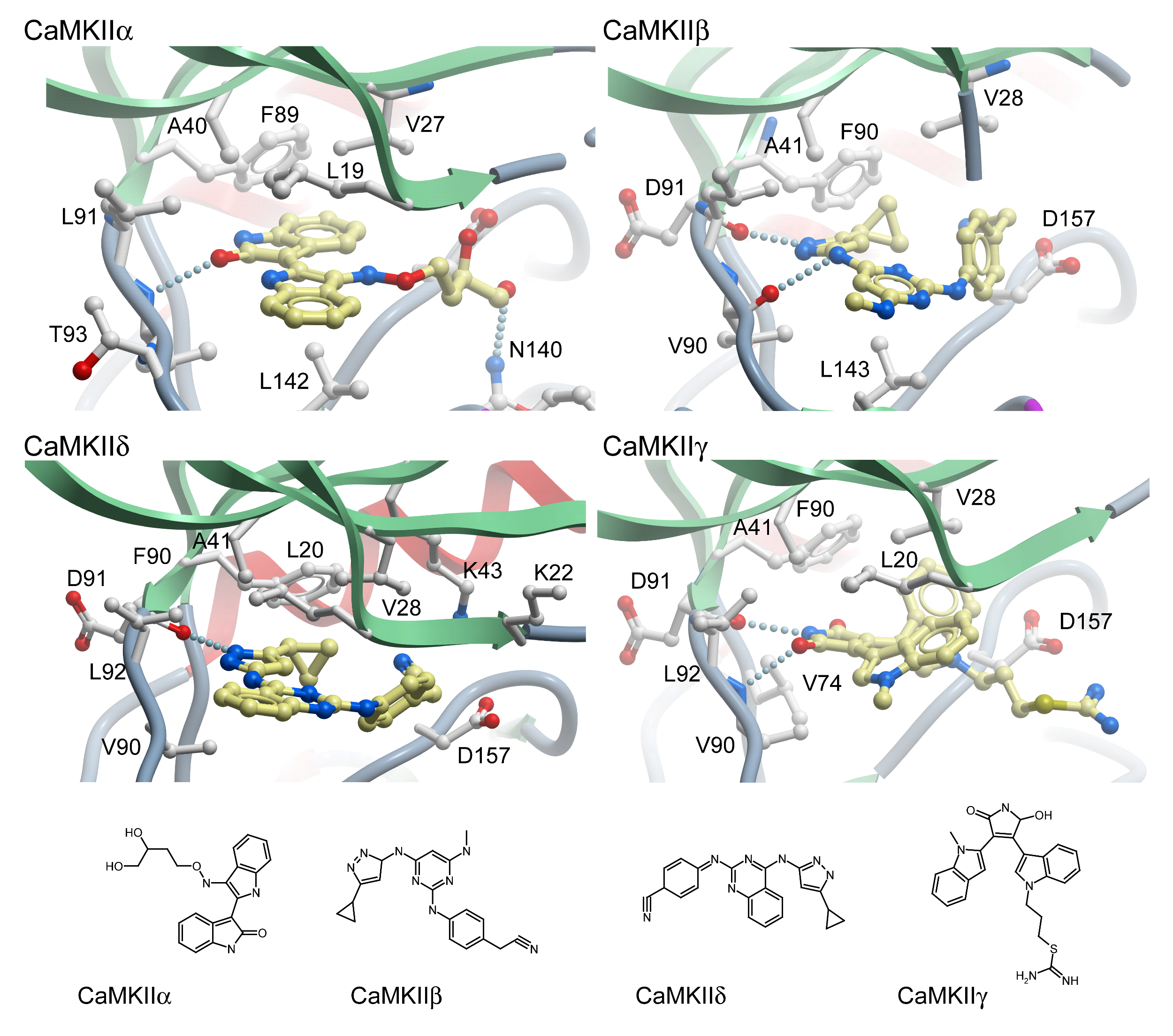

Supplement: Figure S3 — Binding of ATP-competitive inhibitors to the four CaMKII isozymes. The binding mode of each inhibitor is shown in the upper panel and the inhibitor structure in the lower panel. (2.53 MB DOC) [file pbio.1000426.s004.tif]
