# Supplementary material for: Structure of the CaMKIIδ/Calmodulin Complex Reveals the Molecular Mechanism of CaMKII Kinase Activation
Source: PLoS Biol. 2010 Jul 27;8(7):e1000426. doi: 10.1371/journal.pbio.1000426 (PMC2910593; doi:10.1371/journal.pbio.1000426)
Supplement: Table S1 — Structural refinement and data collection. (0.07 MB DOC) [file pbio.1000426.s005.doc]

# **Supplementary Table S1:** Structural Refinement and Data Collection

|  | CaMKIIα | CaMKIIβ | CaMKIIγ | CaMKIIδ | CaMKIIδ / Ca^2+^ / CaM | CaMKIIδ oligom | CaMKIIγ oligom |
| --- | --- | --- | --- | --- | --- | --- | --- |
| PDB code | 2VZ6 | 3BHH | 2V7O | 2VN9 | 2WEL | 2W2C | 2UX0 |
| Ligand | Indirubin E804 | ASC72 | Bisindolylmaleimide IX | ASC65 | SU6656 | – | – |
| Spacegroup | *P*2_1_2_1_2_1_ | *C*2 | *C*222_1_ | *P*3_2_21 | *P*2_1_2_1_2_1_ | *C*2 | *C*222_1_ |
| Cell dimensions [Å]  α, β, γ | *a=*76.24 *b=*113.62, *c=*131.87  90, 90, 90 | *a*=173.40 *b*=79.20 *c*=98.70  90, 104.5, 90 | *a*=84.70 *b*=117.23 *c*=75.97  90, 90, 90 | *a*=*b*=68.28 *c*=313.450  90, 90, 120 | *a*=68.26 *b*=68.85 *c*=121.98  90, 90, 90 | *a*=149.55 *b*=117.80 *c*=160.76  90, 111.92, 90 | *a*=122.95 *b*=158.36 *c*=103.94  90, 90, 90 |
| Crystallization conditions | 12% PEG6K, 0.1M Tris pH 8.5 | 50% PEG 300, 0.2M sodium chloride, 0.1M sodium / potassium phosphate pH 6.2 | 0.1M magnesium chloride, 20% PEG6K, 10% ethylene glycol, 0.1M HEPES pH 7.0 | 1.6M sodium / potassium phosphate, 0.1M HEPES pH 7.5 | 0.1M sodium / potassium phosphate, 20% PEG3350, 10% ethylene glycol | 30% PEG400, 0.1M cadmium chloride, 0.1M sodium acetate pH4.6 | 60% MPD, 0.1 M SPG pH7.0 |
| **Data Collection** |  |  |  |  |  |  |  |
| Resolution [Å] | 2.3 (2.42-2.30)* | 2.4 (2.5 – 2.4) | 2.25 (2.37 – 2.25) | 2.3 (2.42 – 2.30) | 1.9 (2.0 – 1.9) | 2.7 | 2.8 (2.9 – 2.8) |
| Unique observations | 51687 | 50690 | 18326 | 39106 | 45901 | 323492 | 24963 |
| Completeness* [%] | 100 (100) | 99.5 (97.6) | 100 (100) | 100 (100) | 99.7 (100) | 99.7 (100) | 98.3 (90.1) |
| Redundancy* | 4.9 (4.9) | 3.76 (3.65) | 4.0 (4.1) | 4.6 (3.7) | 4.9 (4.9) | 4.6 (4.6) | 3.55 (2.6) |
| *R*_merge_* | 0.123 (0.778) | 0.116 (0.443) | 0.106 (0.722) | 0.089 (0.525) | 0.111 (0.797) | 0.081 (0.761) | 0.054 (0.301) |
| *I* / σ*I** | 11.1 (2.3) | 9.77 (2.69) | 10.40 (1.9) | 10.7 (2.0) | 10.8 (2.6) | 11.6 (2.3) | 12.88 (3.33) |
| Refinement |  |  |  |  |  |  |  |
| Resolution [Å] | 43.15 – 2.3 | 47.78 – 2.4 | 35.62 – 2.25 | 43.03 – 2.3 | 33.34 – 1.9 | 44.8 – 2.7 | 39.68 – 2.80 |
| Reflections (R_free_) | 49347 (2269) | 48113 (2575) | 17388 (936) | 37042 (1960) | 45074 (827) | 68546 (2134) | 23692 (1252) |
| *R*_work_ / *R*_free_ [%] | 16.5 / 20.3 | 22.3 / 27.5 | 17.6 / 24.1 | 21.2 / 24.8 | 16.1 / 19.9 | 21.1 / 24.5 | 21.2 / 23.2 |
| Atoms (P/L/W/O)^#^ | 4674 / 69 / 311 | 8617 / 54 / 262 | 2412 / 51 / 110 | 4721 / 94 / 97 | 3580 / 64 / 405 | 14067 | 6360 / 25/ 99 |
| r.m.s.d bonds [Å]  r.m.s.d angles [°] | 0.015  1.569 | 0.017  1.562 | 0.011  1.269 | 0.014  1.425 | 0.015  1.37 | 0.014  1.362 | 0.013  1.26 |
| Ramachandran^3^  favoured [%]  allowed [%] | 97.2  99.7 | 97.18  100 | 96.3  100 | 97.5  99.3 | 97.7  100 | 97.6  99.9 | 98.3  100 |
| * Values in brackets represent statistics for highest resolution shells.  ^#^ (P/L/W/O): Protein atoms, Ligand atoms, Water, Other. Average *B*-factors include Translation / Libration / Screw (TLS) contributions.  ^3^ Molprobity server analysis (http://molprobity.biochem.duke.edu/) | | | | | | |  |
