# Supplementary material for: Structure of the CaMKIIδ/Calmodulin Complex Reveals the Molecular Mechanism of CaMKII Kinase Activation
Source: PLoS Biol. 2010 Jul 27;8(7):e1000426. doi: 10.1371/journal.pbio.1000426 (PMC2910593; doi:10.1371/journal.pbio.1000426)
Supplement: Table S2 — Dissociation constants for the formation of dimers of CaMK catalytic domains and of “dimers of CaMKII Ca2+/CaM heterodimers” (CaMKII-CaM)2. Protein association was monitored using analytical ultracentrifugation sedimentation velocity experiments, in 10 mM HEPES buffer containing 300 mM NaCl, 1 mM CaCl2 and 5 mM DTT, at 4°C. Distributions of species were analyzed using SEDFIT and dissociation constants were estimated from the proportions between monomer/dimer and heterodimer/heterotetramer peaks, respectively, using K D = c*(m)2/d, where c, concentration in molar; m, proportion of monomers; d, proportion of dimers. (0.05 MB DOC) [file pbio.1000426.s006.doc]

# Supplementary Table S2

Dissociation constants for the formation of dimers of CaMK catalytic domains and of “dimers of CaMKII Ca^2+^/CaM heterodimers” (CaMKII-CaM)_2_. Protein association was monitored using analytical ultracentrifugation sedimentation velocity experiments, in 10 mM HEPES buffer containing 300 mM NaCl, 1 mM CaCl_2_ and 5 mM DTT at 4°C. Distributions of species were analyzed using SEDFIT and dissociation constants were estimated from the proportions between monomer/dimer and heterodimer/heterotetramer peaks, respectively, using *K*_D_=c*(m)2/d, where c, concentration in molar; m, proportion of monomers; d, proportion of dimers.

| Protein | *K*_D_ of CaMKII dimerization of independent kinase domains [µM] | *K*_D_ (CaMKII-CaM) association mediated by the substrate site after Ca^2+^/CaM binding [µM] |
| --- | --- | --- |
| CaMKIIα | 600 | 120 |
| CaMKIIβ | 200 | n.d. |
| CaMKIIγ | 520 | n.d. |
| CaMKIIδ | 490 | 50 |
